# Mosaic quadrivalent influenza vaccine single nanoparticle characterization

**DOI:** 10.1038/s41598-024-54876-2

**Published:** 2024-02-24

**Authors:** Rong Sylvie Yang, Maria Traver, Nathan Barefoot, Tyler Stephens, Casper Alabanza, Javier Manzella-Lapeira, Guozhang Zou, Jeremy Wolff, Yile Li, Melissa Resto, William Shadrick, Yanhong Yang, Vera B. Ivleva, Yaroslav Tsybovsky, Kevin Carlton, Joseph Brzostowski, Jason G. Gall, Q. Paula Lei

**Affiliations:** 1 Vaccine Production Program, Vaccine Research Center, National Institute of Allergy and Infectious Diseases, National Institutes of Health, 9 West Watkins Mill Rd., Gaithersburg, MD 20878 USA; 2 Twinbrook Imaging Facility, LIG, NIAID, NIH, Gaithersburg, MD USA; 3 Vaccine Research Center Electron Microscopy Unit, Cancer Research Technology Program, Leidos Biomedical Research, Inc., Frederick National Laboratory for Cancer Research, Frederick, MD USA

**Keywords:** Fluorescence imaging, TIRFM, Fluorescent labeling, Size-exclusion chromatography, ELISA, Mass spectrometry, Influenza vaccine, Nanoparticle, Biological techniques, Developmental biology, Structural biology

## Abstract

Recent work by our laboratory and others indicates that co-display of multiple antigens on protein-based nanoparticles may be key to induce cross-reactive antibodies that provide broad protection against disease. To reach the ultimate goal of a universal vaccine for seasonal influenza, a mosaic influenza nanoparticle vaccine (FluMos-v1) was developed for clinical trial (NCT04896086). FluMos-v1 is unique in that it is designed to co-display four recently circulating haemagglutinin (HA) strains; however, current vaccine analysis techniques are limited to nanoparticle population analysis, thus, are unable to determine the valency of an individual nanoparticle. For the first time, we demonstrate by total internal reflection fluorescence microscopy and supportive physical–chemical methods that the co-display of four antigens is indeed achieved in single nanoparticles. Additionally, we have determined percentages of multivalent (mosaic) nanoparticles with four, three, or two HA proteins. The integrated imaging and physicochemical methods we have developed for single nanoparticle multivalency will serve to further understand immunogenicity data from our current FluMos-v1 clinical trial.

## Introduction

Influenza viruses can undergo frequent genetic mutations, leading to the emergence of new strains. The current seasonal flu vaccines target specific strains predicted to be prevalent each year. However, this approach requires constant monitoring, updating, and production of new vaccines to match the circulating strains. Development of “universal” vaccines would provide broader and longer-lasting protection against various influenza strains, reducing the need for annual updates.

Most seasonal influenza vaccines target the viral envelope protein Hemagglutinin (HA). There are two major structurally defined antigenic supersites on HA for B cell receptors, one on the globular head region, and the other on the HA stem region^1–9^. The antibodies elicited by the supersites on the HA heads are primarily strain-specific, limited in protection breadth, and have high binding affinity. Alternatively, the stem supersites are conserved among strains, are the targets of cross-reactive antibodies, and do not have as strong affinity. The immunodominance of the HA head results in B cells with limited breadth against divergent influenza strains and represents a major challenge for developing broadly protective influenza vaccines.

Approaches to subvert immunodominance of the HA head domain include chimeric hemagglutinin and mosaic hemagglutinin constructs^10^. The present work focuses on a different approach, a mosaic array of heterotypic influenza HA antigens co-presented on a single nanoparticle^11^. Following vaccination with a mosaic nanoparticle, B cells with cross-reactive B cell receptors capable of bivalent binding to neighboring heterologous antigen pairs can proliferate^1^. Studies also demonstrated that multivalent mosaic nanoparticle vaccines stimulated robust stem-directed antibody responses even in the context of strong pre-existing immunity against the immunodominant HA head^11^. The advantage of mosaic array antigens to enhance broadly neutralizing antibodies provides new possibilities of a broadly protective influenza vaccine.

The heterotypic display of multiple influenza HA antigens is achieved by de novo design of self-assembling proteins^12–14^. Computationally designed two-component nanoparticles have been generated for HA, HIV Env antigens, and RSV proteins to enhance the antigen-elicited antibody titers during the adaptive immune response^15–18^. The FluMos-v1 mosaic nanoparticle vaccine^19^ is comprised of HA proteins from four strains, each with the same dn5B trimerization domain (HA-trimers, abbreviated as HAT) for multivalent surface co-presentation. The HA-trimers and dn5A pentamer proteins (core component of the nanoparticle) were produced and purified individually and assembled into nanoparticles in solution. The successful assembly of monovalent nanoparticles and the presence of four-strain HATs in the nanoparticle population has been demonstrated by EM, immunoprecipitation, mass spectrometry, ELISA, etc.^11^. However, the presence of more than one different HA protein on a single nanoparticle and the distribution of HA proteins have not been determined. The analytical challenge is largely defined by the need to identify more than two components within a 50 nm diameter. Flow cytometry cannot detect the small size of nanoparticles; additionally, the signal intensity arising from small numbers of target proteins is below the limit of detection. The 50 nm diameter size is spatially limiting for antibody + gold particle-labeling with electron microscopy detection. The range of distinguishable gold particle sizes for conjugates (10–100 nm) is too large causing the potential for steric hinderance and an inability to differentiate multiple (four) different HA proteins^20^. To address this issue, we developed a straightforward method that employs labeling each of the four strains of HA-trimers on the FluMos-v1 nanoparticle with distinct fluorophores, applied total internal reflection fluorescence microscopy (TIRFM)^21^ to directly image individual nanoparticles and developed a simple computational method to assess the level of colocalized signal of each of the four HA-trimer types to determine particle valency. Uniquely featured with low background fluorescence, TIRFM enabled the fluorescence detection at the single nanoparticle level and provided direct evidence of multivalency by imaging four HA proteins co-displayed on one nanoparticle via co-localization of four colors. Additionally, we report estimates of the frequency of mosaic nanoparticles formed with two, three, and four different HA proteins. The present work combined standard and innovative techniques to provide new avenues for the characterization of future multivalent nanoparticle vaccines.

## Results

### Characterization of assembled FluMos-v1 nanoparticles

**Figure 1:**
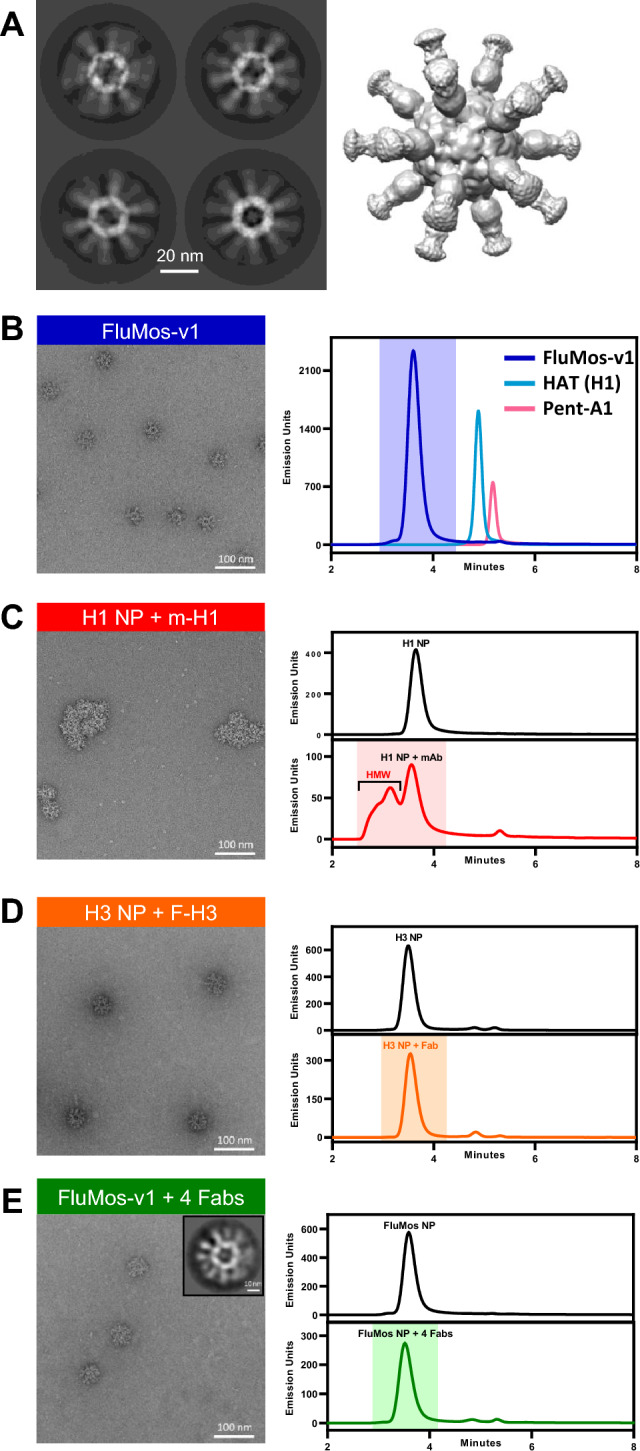
Overview of the structure and assembly of mosaic nanoparticle with influenza hemagglutinin (HA) antigens. (**A**) Imaging of FluMos-v1 using cryo-electron microscopy. Left: 2D class average images showing different views of the highly symmetrical core with long, mobile spikes (HA trimers). Right: 3D reconstruction of FluMos-v1 is shown at a low map threshold to illustrate the HA molecules on the surface of the nanoparticle. (**B**) Negative-stain TEM image (left) of assembled FluMos-v1 nanoparticles and SEC profiles (right) of the nanoparticles (blue) and individual components (pentamer (red), HA trimers (light blue)). (**C**) Negative-stain TEM image (left) and SEC profiles (right) of monovalent H1 nanoparticles. The nanoparticles were combined with the H1 strain-specific monoclonal antibody m-H1 labeled with Dylight405 (TEM and SEC profile, bottom) or analyzed without the antibody (SEC profile, top). (**D**) Negative-stain TEM image (left) and SEC profiles (right) of monovalent H3 nanoparticles. The nanoparticles were combined with the H3 strain-specific Fab fragment F-H3 labeled with Dylight488 (TEM and SEC bottom profile) or analyzed without the Fab (SEC top). (**E**) Negative-stain TEM image (left), representative 2D class average (inset), and SEC profiles (right) of quadrivalent FluMos-v1 nanoparticles. On the right, SEC profiles are the tetravalent FluMos-v1 NP (top) and FluMos-v1 mixed with labeled Fabs.

The ~ 30 nm diameter Flumos-v1 nanoparticle vaccine is composed of 20 HA-dn5B trimers and 12 dn5A pentamers with four strains of influenza Hemagglutinin (H1, H3, HBv, and HBy) to display heterotypic HA trimers on the surface of the nanoparticle. The successful assembly of the influenza vaccine nanoparticle is demonstrated by electron microscopy and SEC. Cryo-EM 2D class averages and a 3D reconstruction at a resolution of 4.9 Å (Fig. 1A, left and right, respectively) confirm the symmetric configuration of the nanoparticle which is consistent with its icosahedral design. The particle’s symmetry suggested there would not be positional bias for Fab binding. The molar ratios of the four HA-trimers assembled into FluMos-v1 nanoparticles were predicted to be 1:1:1:1 based on the assembly condition of equimolar addition of the HA trimers. We previously experimentally confirmed the molar ratio by ELISA^19^ using four strain-specific monoclonal antibodies. For this study, we determined the molar ratio with three of the same monoclonal antibodies and with the MM09 antibody to HBy (Supplementary Figure S2) to be 1.0:1.1:1.1:1.0, consistent with equal molar distribution of the four HATs in FluMos-v1.

A key consideration for single nanoparticle evaluation was the assessment of mono-dispersity of nanoparticles in solution. Negative-stain TEM of assembled FluMos-V1 nanoparticles revealed no aggregates, consistent with the SEC chromatogram (Fig. 1B). Additionally, the largest molecular weight peak corresponded to single nanoparticles, demonstrating the absence of aggregated particles. In contrast, solutions of nanoparticles bound to IgG monoclonals contained substantial amounts of aggregates which were evident as a broad high molecular weight peak in the SEC chromatogram (Fig. 1C). Monovalent HA nanoparticles bound to the Fab fragment of the IgG were monodisperse in TEM images, with no detectable high molecular weight SEC peak corresponding to aggregates of particles (Fig. 1D). Additionally, samples of quadrivalent FluMos-v1 nanoparticle with the four corresponding HA Fabs were 91% pure and without aggregates (Fig. 1E). Characterization of FluMos-V1 by negative-stain electron microscopy, SEC, and ELISA indicated that Fab binding did not induce aggregation and there was equimolar population-wide incorporation stoichiometry of the four HA proteins.

### Characterization of Fab Fluorescent labeling by mass spectrometry

To confirm multivalency of FluMos-v1 by microscopic imaging, the mAb binding partners of HA strains were chemically labeled with CF-405S, Dylight 488, Dylight 560, or Dylight 650 and enzymatically cleaved to generate fluorescently labeled Fabs. Using Fabs instead of IgGs mitigates potential for nanoparticle aggregation caused by the interaction of bivalent arms from one IgG with two nanoparticles. Labeling efficiency of the Fab fragments ranged from 10 to 75% as determined by MALDI-MS, and the majority of labeled Fabs carried one fluorophore (Table 1, Supplementary Figure S3). F-HBy was the least efficiently labeled Fab, with 10% of molecules having one label and 90% unlabeled. Therefore, most of the F-HBy Fab fragments binding to the nanoparticle will not have the fluorescent label, likely leading to inefficient detection of the B-Yamagata strain HA-trimer. Alternatively, Dylight 488 was used to label all four Fabs, with MALDI-MS analysis showing unlabeled Fabs and Fabs with up to three Dylight 488 tags for all four Fabs (Figure S4).

**Table 1:** The relative abundance (percentage) of FLR labeled and unlabeled Fab determined by MALDI-MS and calculated from peak height (intensities).

Sample	Number of dye molecules			
	0	1	2	3
CF405S on F-H1	46	38	15	1
D550 on F-H3	37	49	13	n.d
D488 on F-BV	25	56	17	2
D650 on F-BY	90	10	n.d	n.d

*n.d., not detected.

### Confirmation of equimolar content of the four HA trimers by SEC-fluorescence detection

**Figure 2:**
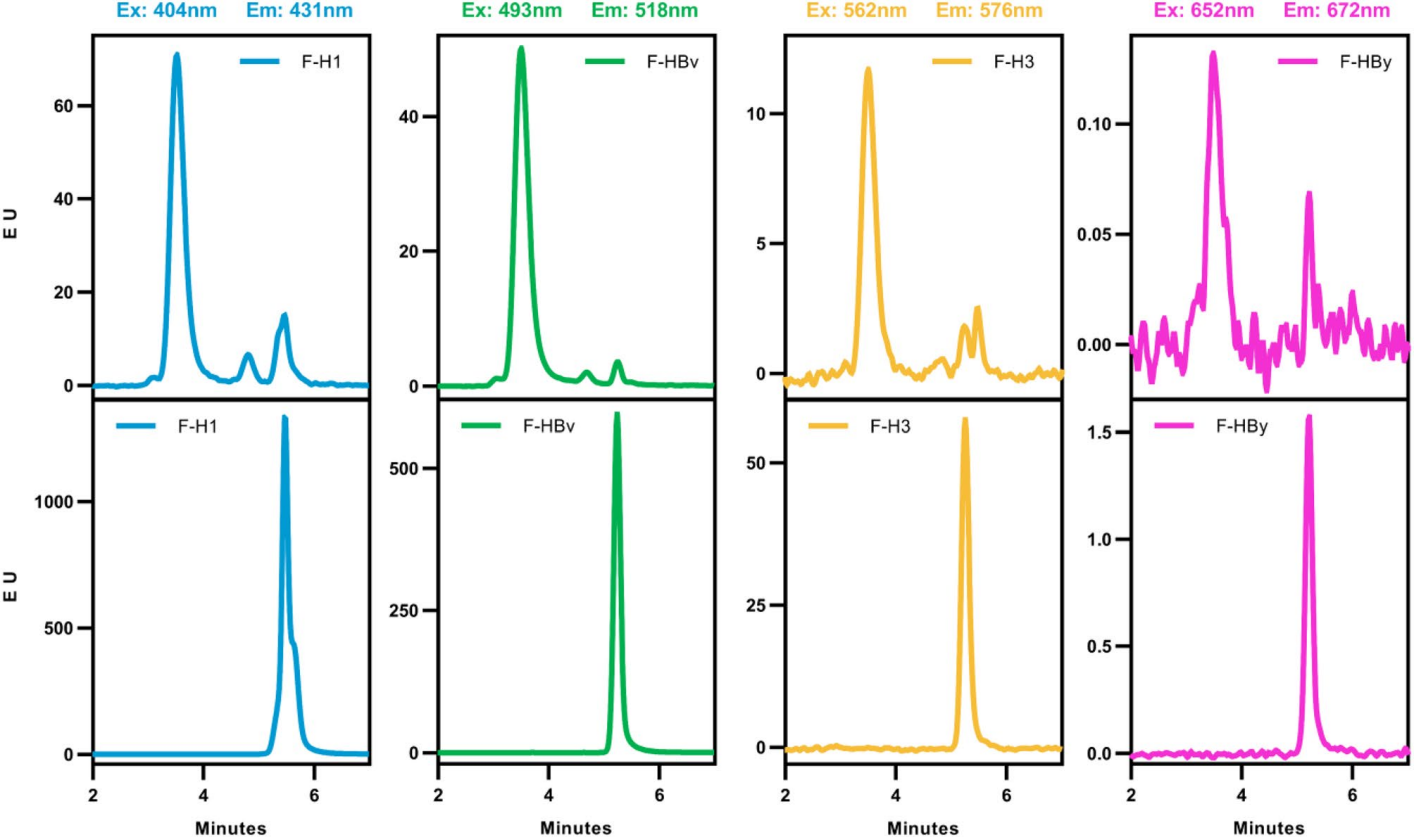
SEC Analysis of FLR-Fab Labeling of the FluMos-v1 nanoparticle. FluMos-v1 labeled with a mixture of the four Fabs (F-H1, F-HBv, F-H3, and F-HBy) (top) or the four Fab mixtures without FluMos-v1 (bottom) were injected into the SEC column. Each chromatogram represents a different fluorescent channel corresponding to the FLR-Fab-specific fluorescent label as noted above each pair or chromatograms.

**Figure 3:**
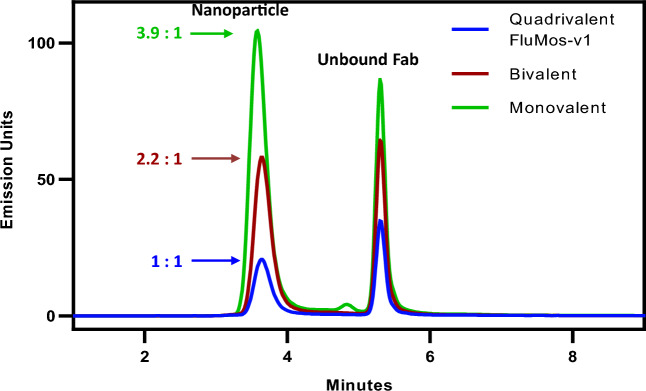
SEC-FLR of DyLight-488 F-H3 and nanoparticles assembled with the H3 HA-trimer (Monovalent), the H3 and H1 HA-trimers (Bivalent), or all four HA-trimers (quadrivalent FluMos-v1). Peak area ratios are noted by the arrows and represent the fluorescence signal intensity ratio of Fab-H3 detected in monovalent (green), bivalent (red) and quadrivalent (blue) nanoparticles, with the 1:1 ratio defined as the Fab-H3 peak area of the quadrivalent nanoparticle.

The utility of fluorescently labeled Fab fragments for detection of HA proteins incorporated into nanoparticles was determined by an SEC-fluorescence (SEC-FLR) method. Equal molar amounts of CF405S-F-H1, D550-F-H3, D488-F-HBv, and D650-F-HBy were pre-mixed, incubated with FluMos-v1, and buffer-exchanged into FluMos-v1 formulation buffer. The FLR-Fab and FluMos-v1 combination was analyzed in separate SEC runs at the excitation/emission wavelength of each fluorophore (Fig. 2). The main peak at retention time ~ 3.5 min of the FluMos-v1/FLR-Fab complex confirms the presence of each fluorescently labeled Fab predominantly in complex with the nanoparticle. Fabs not bound to FluMos-v1 are evident as minor peaks. The SEC chromatograms of nanoparticle/FLR-Fab complexes confirm that the nanoparticles were labeled with the four fluorophores. Additionally, the largest molecular weight peak corresponded to single nanoparticles + Fabs, demonstrating the absence of aggregated Fab/nanoparticle complexes. As expected from the low labeling efficiency, the fluorescence intensity of D650-F-HBy was much lower than that of the other FLR-Fabs. Next, the molar ratio of H1, H3, HBv, and HBy was determined by SEC-FLR of nanoparticles bound to single D488-labeled Fabs. The theoretical maximum number of Fab binding sites for a single strain of HAT per nanoparticle are 60, 30, and 15 for monovalent, bivalent, and quadrivalent nanoparticles, resulting in a ratio of 4:2:1 if each strain was incorporated equally. For the determination of HA molar ratio, individual strain D488-labeled Fabs were complexed with monovalent, bivalent, and quadrivalent nanoparticles at Fab-to-nanoparticle ratios of 60:1, 40:1, and 20:1, respectively. Fabs were added in slight excess to saturate the epitopes on the nanoparticle while establishing conditions for subsequent measurement, as peaks corresponding to unbound Fabs were apparent in a preliminary experiment at Fab-to-nanoparticle ratios of 60:1, 40:1, and 20:1 (Supplementary Figure S5). The SEC-FLR chromatogram illustrates the fluorescence signal peaks (Ex493/Em518 nm) of Dylight 488-F-H3 complexed with H3 monovalent, H1/H3 bivalent, and H1/H3/HBv/HBy quadrivalent nanoparticles (Fig. 3). Experimental ratios of H3 HA-trimer in the nanoparticle samples were determined by dividing the normalized peak area of each nanoparticle by the normalized peak area of the quadrivalent nanoparticle. The FLR peak intensity ratios for the complexes of Fab with H3 monovalent, H1/H3 bivalent, and H1/H3/HBv/HBy quadrivalent nanoparticles were 3.9:2.2:1. The peak intensity ratios of the other three strains (H1, HBv, HBy) were approximately 4:2:1, with slight deviation for the HBy at 5:2:1 (Supplementary Table S1). The consistency of peak intensity ratios among H1, H3, HBv, and HBy demonstrates equal molar distribution of the four HA strains in the preparation of FluMos-v1, converging with the results obtained by LC-MS^22^ and ELISA^19^. The agreement between the three methods demonstrates the suitability of fluorescently labeled Fabs for HA detection.

### *Demonstration of multivalent individual nanoparticles *via* TIRF microscopy*

**Figure 4:**
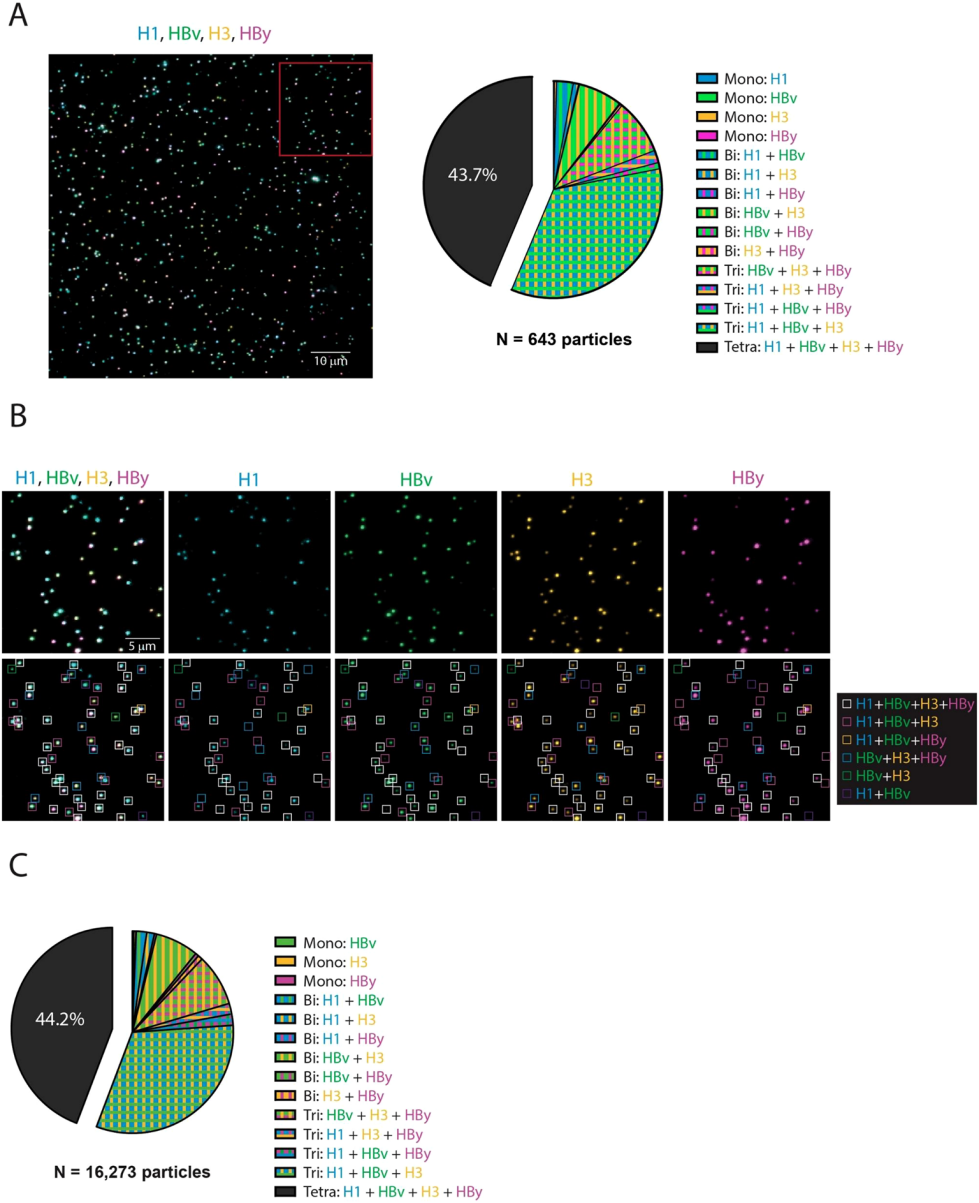
FluMos-v1 nanoparticles exhibit a high degree of multivalency. (**A**) FluMos-v1 nanoparticles were labeled with fluorescent Fab fragments corresponding to each of the four hemagglutinin strains, then diluted in formulation buffer and imaged via TIRF microscopy. One field of view (FOV) is shown, representative of twenty random fields imaged. (**B**) Top row: red inset from (**A**) is expanded and separated by channel. Bottom row: individual nanoparticles are categorized by valency according to NIS-Elements-based analysis program. (**C**) Percentage of FluMos-v1 nanoparticles from all twenty FOVs in each valency category as determined by NIS-Elements-based analysis program.

**Figure 5:**
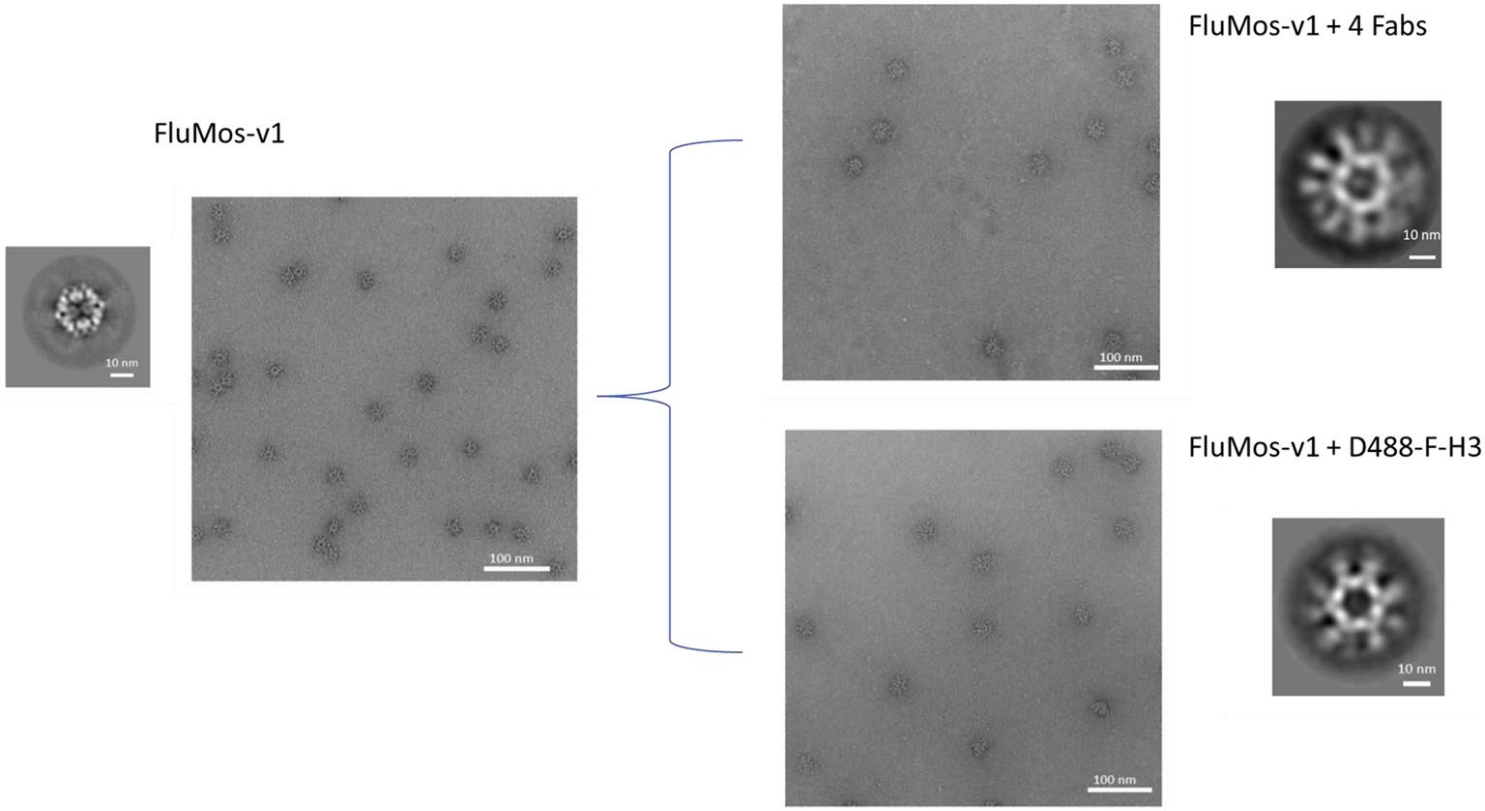
Negative-stain EM of FluMos-v1, FluMos-v1 complexed with four unlabeled Fabs, and FluMos-v1 complexed with D488-F-H3. Representative micrographs are shown along with representative 2D class averages.

After confirming that FluMos-v1 nanoparticles were labeled with fluorescent Fab fragments, and that the overall population of quadrivalent assembled FluMos-v1 nanoparticles has roughly equivalent molar ratios of each HA trimer (Figs. 1D,E, 3, and^22^), the fluorescently labeled FluMos-v1 nanoparticles were next visualized by total internal reflection fluorescence microscopy (TIRFM) to demonstrate multivalency at the individual nanoparticle level. TIRFM is a well-established epi-fluorescence imaging technique that is used to excite fluorescent molecules in aqueous solution proximal (within ~ 100 nm) to the glass coverslip of an imaging chamber^23^. Because small fluorescent molecules, including unbound labeled Fab, suspended in solution are not excited by TIRFM, extremely high-contrast images of individual FluMos-v1 nanoparticles adhered to the glass coverslip could be captured and evaluated for multivalency. A solution of FluMos-v1 nanoparticles with each HA trimer targeted by the fluorescent Fab fragments was placed in wells with glass coverslip bottoms and imaged using a through-objective, inverted TIRF microscope and EMCCD camera (Fig. 4A). The majority of fluorescent spots observed in TIRFM images were considered individual nanoparticles based on the high degree of monomeric purity of the sample used for fluorescence imaging as shown by SEC of nanoparticles bound to the Fabs (Fig. 2) and further illustrated by negative stain EM. The latter method demonstrated that similarly prepared samples, although greater than 1000-fold more concentrated than for TIRFM, of FluMos-v1 or FluMos-v1 labeled with the four Fabs or D488-F-H3 appear as ~ 30 nm diameter dispersed monomeric nanoparticles with, at most, two to three nanoparticles in close proximity (Fig. 5). 2D classification of unlabeled FluMos-v1 nanoparticles and nanoparticles labeled with one and four Fabs produced progressively less well defined 2D class averages, reflecting increasing structural heterogeneity associated with Fabs binding to HATs of four different types randomly distributed on symmetrical nanoparticle cores. Importantly, individual HATs not complexed with nanoparticle cores were not observed by negative-stain TEM. The validity of HA-specific signal vs background was established by imaging the control samples containing singly- or doubly- labeled nanoparticles (Supplementary Figure S5). No signal was observed in channels where the HA trimer was not labeled. TIRFM demonstrated that individual quadrivalent assembled FluMos-v1 nanoparticles were observed with multiple colors, confirming that the population was indeed multivalent, but with varying fluorescent intensities. Because the Fab fragments used to target each of the four HA trimers carried a range of fluorescent dye molecules (Table 1), limited conclusions can be drawn from the observed intensity differences between nanoparticles in TIRFM images (Fig. 4A,B).

To analyze nanoparticle valency, we developed an algorithm to categorize the colors of more than sixteen thousand individual FluMos-v1 nanoparticles in the population imaged by TIRFM (Fig. 4C). Localizations were established via local maxima determination above a threshold set by examination of singly- and doubly labeled control nanoparticles (Supplementary Figure S5). Localizations with weak signal intensity in any given channel were included in the analysis if a fluorescent signal was detected for at least one other color in the same localization. However, localizations of only one color with weak signal intensity were assumed to be dissociated HA trimers and were excluded from the analysis, as true monovalent nanoparticles should be of greater intensity relative to multivalent nanoparticles with the same color.

Impressively, TIRFM image analysis (Fig. 4C) revealed that a plurality (44.2%) of the FluMos-v1 nanoparticle population is indeed quadrivalent, with a nearly equivalent percentage (43.7%) of trivalent nanoparticles of varying types (percentage of each population of nanoparticles is summarized in Table S2 corresponding to Fig. 4A and C). For trivalent nanoparticles (those lacking signal from one fluorescently labeled Fab), H3-HBv-HBy, H1-HBv-HBy, H1-H3-HBy, and H1-H3-HBv made up 8.3%, 1.8%, 1.7%, and 31.9% of the total population respectively. It is important to highlight that the largest percentage of trivalent nanoparticles (31.9%) were those lacking signal from F-HBy Fab fragment (magenta). As stated, the highest level of fluorescent labeling obtainable for F-HBy was 10% (Table 1), indicating that the true number of quadrivalent FluMos-v1 nanoparticles was likely undercounted. Three independent preparations of quadrivalent assembled FluMos-v1 nanoparticles imaged by TIRFM showed an average measurement of 43.7% (2.9% STD) nanoparticle/Fab complexes excitable at all four fluorescent wavelengths.

## Discussion

### General conclusions

Our study demonstrates that FluMos-v1 nanoparticles are predominantly quadrivalent. Imaging by TIRFM allowed direct visualization of the co-localization of HA trimers from multiple strains, and imaging of more than 10 thousand nanoparticles revealed individual nanoparticles with four, three, two and one color. In support of the single nanoparticle visualization by TIRFM, electron microscopy and SEC provided clear evidence for single-particle dispersion in solution, thus excluding the possibility of the multi-color TIRFM species corresponding to aggregates of nanoparticles. Color distribution analysis of TIRFM data of these nanoparticles demonstrated that 44% are quadrivalent nanoparticles. As Fab dye-labeling efficiency differed for the four Fabs, and fluorescently labeled and unlabeled Fabs were equally capable of binding to the HAs on FluMos-v1 (Supplementary Figure S1), the population of 43.6% of tri-labeled nanoparticles likely includes quadrivalent nanoparticles wherein the fourth HA was not labeled with a fluorescently tagged Fab (MALDI-MS data for the labeling efficiency in Supplementary Figure S3). In particular, the proportion of tri-labeled nanoparticles lacking signal for the Hby HA, 31.9%, is substantially higher than the proportion of other tri-labeled nanoparticles, ranging from 1.8 to 8.3%. The D650-F-Hby Fab was labeled at the lowest efficiency of all the Fabs, at only 10%, whereas the other Fabs were labeled with greater than 50% efficiency (MALDI-MS data for the labeling efficiency in Supplementary Figure S3). Taken together, it is likely that a significant proportion of the 31.9% of tri-labeled nanoparticles lacking signal from the labeled Hby Fab were bound to unlabeled Hby Fab and the actual proportion of quadrivalent nanoparticles is significantly greater than the measured 44%.

The assembly of FluMos-v1 as a 120-subunit icosahedral complex is a highly cooperative process^24^ driven by non-covalent interactions of two constituent protein building blocks, HA-dn5B trimer and dn5A pentamer. Previous work^24^ demonstrated that assembly process with multiple HA-trimers was more efficient than for monovalent particles. For example, Hby- and HBv- trimers had higher assembly efficiencies into heterologous nanoparticles in the presence of H1- or H3-trimers. Thus, the estimate that a minimum of 44% of individual nanoparticles had all four HA-dn5B trimers is consistent with these observations on assembly efficiency. The spatial distribution and copy number of the four HA components on nanoparticles remain to be determined. However, our analysis of single particles showed that in the population of Flumos-v1, nearly 90% of nanoparticles are quadrivalent and trivalent.

### Considerations for other imaging and detection technologies

Given the increased use of nanoparticles as vaccine vehicles to deliver antigens to induce a potent long-lasting immune response^11^, it would be of interest for production laboratories to easily and rapidly appreciate antigen valency at the level of individual nanoparticles to complement a population analysis. To this end, we asked a very simple question: Can the valency of an individual nanoparticle be assessed by a fluorescence imaging technique, namely TIRFM, that is straightforward enough to work in the hands of a researcher with only general imaging experience? The techniques we employed allowed us to determine that quadrivalent nanoparticles were assembled through a simple co-localization imaging experiment that required about an hour of imaging time and a similar amount of time for analysis. While the objects we imaged were indeed diffraction-limited, we did not require any specialized super-resolution methodology for acquisition or analysis. Our method accommodates the need for rapid and robust analysis, while quantifying how many HA trimer species reside on a single multivalent nanoparticle is beyond our scope.

SRM encompasses a variety of fluorescence imaging techniques that enable investigators to break the so-called diffraction-limit of optical microscopy, thereby, allowing objects that could only be resolved at ~ 300 nm in x–y space by standard imaging modalities to be discriminated in space at distances of ~ 120 nm down to 10 nm depending on the technique used. Excellent reviews can be found on how these techniques work and the types of questions that can be addressed with them^25–27^. Clearly, it is beyond the scope of this article to make direct comparisons with these techniques; however, it is critical to highlight that SRM techniques have been eloquently employed to study protein localization on viral particles and nanoparticles, providing interesting insights into protein localization and interpretations of function^28–31^. A main limitation of these techniques is the expertise and specialized equipment/reagents required for their implementation; furthermore, it is important to note that certain SRM fluorescence techniques are limited in the number or type of fluorophores that can be easily used in a single experiment, which would be prohibitive to employ in this present study. In the same vein, specialized techniques like fluorescence lifetime imaging (FLIM), Raman spectroscopy and cryo-electron microscopy (cryo-EM) offer potential alternative modalities to study the composition of self-assembling nanoparticles.

FLIM measures the time a fluorophore persists in an excited state before releasing a photon that is, in turn, counted by a specialized detector to produce a lifetime decay curve^32,33^. The lifetime of a fluorophore is a unique signature and can be used to discriminate the presence of different colored fluorophores in space (as in an image). However, fluorescence lifetime can be affected (decreased) by quenching due to a chemical change or the proximity of another fluorophore such as seen in Forster resonance energy transfer—which is a real consideration for nanoscale objects. While FLIM is a modality that potentially can identify multiple fluorophores of varying colors on a single nanoparticle, detecting single particles is still necessary and would likely need to be coupled with total internal reflection illumination to provide the necessary contrast, a coupling which is technologically challenging and has rarely been implemented^34^.

Raman spectroscopy encompasses a variety of methodologies that are used to fingerprint biological molecules. The technique is based on the principle that when light is incident on molecules (in solution or on a substrate) it scatters both elastically (Rayleigh scattering), maintaining its frequency, or inelastically, where the frequency (and energy) of the scattered light either decreases (Stokes Raman scattering) or increases (Anti-Stokes Raman scattering). While the Raman signal produced is tiny and needs to be filtered away from the dominant Rayleigh scattered light, it is unique, thus providing an identifiable signature for the molecules under study. Raman spectroscopy has been used to characterize viral proteins, diagnose the presence of viruses in cells, and to study the viral life cycle^35^. One technique in particular, tip-enhanced Raman spectroscopy (TERS) has been used to discriminate viral types^36^ and may be an intriguing alternative method to determine the valency of FluMos-v1 and similar nanoparticulate vaccine vehicles. TERS employs a probe that is scanned across a sample supported on a flat substrate analogous to how a cantilevered tip is used in atomic force microscopy. The probe, thereby, provides spatial resolution on top of the Raman signal and has been used successfully to differentiate flu from coxsackie virus^36^.

While highly specialized, requiring advanced technical skills and equipment, Cryo-EM is another potential methodology for determining nanoparticle valency. Cryo-EM has advanced significantly over recent years and improved methods in sample preparation, equipment and data analysis/artifact removal have made it possible to exquisitely visualize the fine features of viruses and even biomolecules^37,38^. If the 3D structure of each HA trimer can be distinguished by this technique, it would then eliminate the time-consuming process of developing and labeling quality anti-HA monoclonal antibodies for fluorescence microscopy. However, the expertise and expense required by this method remains a consideration.

### Significance

In conclusion, the major advantage of a mosaic influenza nanoparticle vaccine over cocktails of monovalent nanoparticles is the broadly neutralizing activity against multiple viral strains by elicitation of cross-reactive B-cells to generate cross-reactive antibodies. The demonstration of predominantly multivalent particles in the FluMos-v1 vaccine is critical for supporting the concept of a mosaic vaccine rather than a mixture of monovalent or bi-valent nanoparticles. The methods employed here, SEC, ELISA, electron microscopy, and total internal reflection fluorescence microscopy (TIRFM), are broadly applicable to any complex nanoparticle approach for vaccines and therapeutics. The strategies developed in this study not only shed light on the characteristics of the assembly at the single nanoparticle level but will help further our understanding of the relationships between the structure and the clinical trial data (NCT04896086).

## Material and methods

### Abbeviation

Influenza strains, monoclonal antibodies (mAb), F(ab) fragments (Fab), and their abbreviations are in Table 2.

**Table 2:** Influenza strains and antibodies used in the study.

Influenza strain		Anti-HA mAb		
Name	Subtype	Name	Abbreviation	F(ab)
A/Idaho/07/2018	H1	315-02-1H01	mH1	F-H1
A/Perth/1008/2019	H3	315-24-1E07	mH3	F-H3
vic-B/Colorado/06/2017	HBv	R95-1E12	mHBv	F-HBv
Yam-B/Phuket/3073/2013	HBy	MM09 mAb	mHby	F-HBy

### Materials

Quadrivalent FluMos-v1, monovalent, bivalent, and trivalent nanoparticles^39^, HA-trimer, Pentamer, and their formulation buffers were produced/prepared at the VPP. Monoclonal antibodies m-H1, m-H3, m-HBv, and m-HBy (1B01) were obtained from the VRC, and another m-Hby was purchased from Sino Biological (Catalog number 11053-MM09). Dylight fluorescent labeling kits were purchased from Thermo Scientific (DyLight Antibody Labeling Kits, Thermo PI53024). CF405-S Fluorescent labeling kit was purchased from Biotium (Catalog number 92211). FabALACTICA Fab kits were purchased from Genovis (AK-AFK-025). Amicon Ultra centrifugal filters were purchased from Millipore (Ultracel 100k, UFC510096). Sodium phosphate monobasic (monohydrate) and sodium phosphate dibasic (heptahydrate) was purchased from JT Baker (Catalog number 3802-01, and 3803-01, respectively).

### Methods

*Fluorescence labeling with DyLight fluorophores.* mAbs were chemically labeled with fluorophore DyLight 488, DyLight 550, and DyLight 650 by adding 40μL of the borate buffer (0.67 M) to 0.5 mL of mAb solution at 2 mg/mL in PBS. Then 0.5 mL of the prepared mAb were added to the vial of DyLight reagent, vortexed and briefly centrifuged to collect the sample in the bottom of the tube. The reaction mixture was incubated for 60 min at room temperature with protection from light.

*Fluorescence labeling with CF405S.* mAb 1H01 was chemically labeled with fluorophore CF405S. 100 µL of 1 M sodium bicarbonate pH 8.3 is added to the 900 µL of 1.0 mg antibody solution. A vial of CF405S dye was equilibrated to room temperature, and then 25 µL anhydrous DMSO was added to the dye vial. The vial was vortexed and then briefly centrifuged to collect the dye solution at the bottom of the vial. The dye stock was then mixed with the antibody solution prepared in the first step. The antibody/dye solution was protected from light by wrapping in aluminum foil. The reaction mixture was incubated for 1 h at room temperature with gentle rocking. The labeled protein was stored at 4° C in the dark.

*Fab fragment generation.* Fluorescently labeled mAbs were cleaved above the hinge region by IgdE enzyme to generate the DyLight- and CF405S-labeled Fabs. The fluorescently labeled mAbs were buffer exchanged into 150 mM sodium phosphate buffer pH 7.0 using 7 kDa MWCO Zeba column (Thermo Scientific catalog number 89882). The digestion was performed at room temperature overnight. After incubation with immobilized FabALACTICA resin the fragments were then collected by centrifugation and buffer exchanged into PBS buffer (10 mM sodium phosphate, 150 mM NaCl, pH 7.4). The Fab fragments were subsequently separated from the Fc by incubating the digest using the CaptureSelect™ Fc column(s) with multi-species Fc affinity resin at room temperature for 30–45 min. The pure Fab fragments were then collected by a centrifugation step. Fluorescent labeling did not change the binding profiles of the Fabs or their EC-50 values (Supplementary Figure S1).

*SEC (Size exclusion chromatography).* The samples were loaded onto an SEC column (Sepax, SRT SEC-1000 PN: 215950-7815, 7.8 × 150 mm, 5 um) and analyzed by UPLC (Waters Acquity H-Class) with fluorescence (Ex278/Em330, Ex404/431, Ex493/516, Ex562/576, Ex652/672 nm) and UV (280 nm, 215 nm) detection. Mosaic nanoparticles complexed with Fabs or fluorescently labeled-Fabs were eluted by 2xPBS in an isocratic gradient over 12 min, at a flow rate of 1 mL/min. For SEC-FLR, the total areas of the main peak of the nanoparticle + florescent-Fab complex were determined by Empower software. To calculate the ratios of peak areas for the nanoparticle in complex with the D488-labeled Fabs, the area under the main peaks at the D488 channel (Ex493/Em518 nm) and intrinsic fluorescence channel (Ex278/Em330nm) were separately integrated. The main peak areas at 493/518 nm were multiplied by a normalization factor determined from the intrinsic fluorescence peaks (Eq. 1) to compensate for differences in nanoparticle concentrations loaded onto the SEC column.

Equation 1: Normalization factor.

1 $${\text{f}} = \frac{{{\text{Main Peak Area}}_{{{\text{Ex278}}/{\text{Em33}}0}} {\text{of monovalent nanoparticle}}/{\text{Fab complex}}}}{{{\text{Main Peak Area}}_{{{\text{Ex278}}/{\text{Em33}}0}} {\text{of the selected nanoparticle}}/{\text{Fab complex}}}}$$

*Fab – nanoparticle complex formation.* For monovalent, bivalent, and quadrivalent influenza nanoparticles, the Dylight/CF405S-labeled Fabs were incubated with the nanoparticle on a Jitterbug-4 benchtop shaker (Boekel Scientific, Catalog number 270440) for 30 min, at 350 rpm and 38 °C. Samples were then centrifuged at 11,000 rpm, 2–3 min, for 3 times with Amicon Ultra 100 k centrifugal filters to remove unbound fluorescent-Fabs and buffer exchanged into formulation buffer (20 mM Acetate Phosphate, 280 mM NaCl, 5% sucrose, 2.5% sorbitol, 0.01% PF68, pH 5.7) or 1xPBS buffer. For samples tested in the SEC-fluorescence detection method for confirmation of equimolar content of the four HA trimers, no centrifugation step was performed.

*ELISA.* Nunc MaxiSorp plates (VWR, Catalog 62409-024) were coated with 10 µg/mL of Lectin from *Galanthus nivalis* (snowdrop) (Millipore Sigma, Catalog L8275) and incubated at 4 °C overnight. FluMos-v1 nanoparticle samples were serially diluted in fourfold steps and added to the wells for 1 h at 37 °C with shaking. Strain-specific mAb/Fab prepared at 2 µg/mL was added to each well and incubated for 1 h at 37 °C with shaking. Horseradish peroxidase (HRP)-conjugated anti-human (Jackson ImmunoResearch, Catalog 109-035-003), prepared at 1:60,000 dilution, was added and incubated at room temperature for 1 h with shaking. The color product was developed with 3,3′,5′,5-tetramethylbenzidine (Ultra-TMB) substrate (ThermoFisher Scientific, Catalog 34028), and reaction was stopped by adding TMB stop solution (ThermoFisher Scientific, Catalog N600). The optical signal was measured by absorbance at 450 nm with a Spectramax plate reader (Molecular Devices, MV 02861).

*Reverse phase liquid chromatography (RPLC).* Mobile phase A (MPA) was composed of LC–MS grade water and 0.1% trifluoroacetic acid (TFA) (Thermo, 28904). Mobile phase B (MPB) was composed of 75% LC–MS grade acetonitrile (Burdick & Jackson, LC015-1), 20% Isopropanol (IPA) (J.T. Baker, 9083-01), 5% water, and 0.085% TFA. The gradient (0 min, 38% B; 2 min, 38% B; 12 min, 40% B; 17 min, 40% B; 18 min, 45% B; 22 min, 45% B; followed by column wash) was performed on a Waters Acquity UPLC Protein BEH C4, 300A, 1.7 µm, 2.1 × 100 mm column. The method was run at 0.2 mL/min, at a column temperature of 80 °C, and a sample temperature of 8° C. The nanoparticles were diluted to 0.12 mg/mL in PBS and 25 µL was injected (3 µg). Due to the denaturing property of the organic mobile phase, the nanoparticles are disassembled immediately after injection, and various HA molecules elute in separate peaks in the order of increasing hydrophobicity. The signal was monitored by UV at 280 nm. To calculate the molar ratio of various HA strains, the chromatographic peak areas at the detection wavelength of 280 nm were divided by the extinction coefficient of the corresponding HAs (Extinction coefficient of H1, H3, HBv and HBy are 1.68, 1.47, 1.22, and 1.26, respectively. The extinction coefficient of 1.24 is used for co-eluting B strains).

*MALDI-MS.* A microFlex LT MALDI-TOF mass spectrometer (Bruker Daltonics, Bremen, Germany), run in positive mode with a pulsed nitrogen (emission 337 nm), 1000 ns delay, and 2 kV accelerating voltage, was used to measure the mass of the intact fluorescently labeled mAbs and Fabs. One µL of the purified sample (2 mg/mL) was mixed with 1 µL of 0.1% TFA and 1 µL of 10 mg/mL sinapinic acid solution (Ricca Chemical, MB15103241C) in 50% acetonitrile. One µL of the mixture was deposited onto the stainless steel MALDI target plate, air-dried, and measured by MALDI-MS. The data were collected with flexControl and processed with flexAnalysis (Bruker Daltonics).

*Cryo-EM*. FluMos-v1 quadrivalent nanoparticles at a concentration of ~ 0.2 mg/mL were vitrified at room temperature and 95% humidity using Vitrobot Mark IV (FEI, Netherlands) by applying a 2.7-µl drop on a holey carbon grid (Quantifoil R 1.2/1.3) covered with a monolayer of graphene oxide and, after blotting, plunging the grid into liquid ethane. Data was collected using a Thermo Scientific Krios G1 electron microscope operated at 300 kV. 970 movies were recorded using a Falcon 3EC direct electron detector in the counting mode at a pixel size of 1.44 Å. The defocus range was 1–3 µm underfocus. Motion correction was performed with MotionCor2^40^. Ctffind 4.1 was used to estimate the defocus of motion-corrected micrographs^41^. 2D classification and 3D reconstruction were performed with Relion 3^42^. The resolution of the final map, determined from a dataset containing 16,416 particles with imposed icosahedral symmetry, was 4.9 Å.

*Negative Stain Electron Microscopy.* Nanoparticle samples were diluted to about 0.05 mg/mL with buffer containing 10 mM HEPES, pH 7.0, and 150 mM NaCl. Nanoparticles were adsorbed to a glow-discharged carbon-coated copper electron-microscopy grid (Electron Microscopy Sciences) by applying a 4.7-µl drop of the diluted sample for 15 s and removing the drop with filter paper. This was followed by three washes with the above buffer conducted in the same way. After the final wash, adsorbed material was negatively stained by applying consecutively three drops of 0.7% uranyl formate in the same manner and allowing the grid to air-dry. Datasets were collected on a Thermo Scientific Talos F200C transmission electron microscope operated at 200 kV and equipped with a Ceta camera. Particle picking was performed automatically using in-house written software (Y.T., unpublished). Relion 3^42^ was used for reference-free 2D classification.

*Measuring Multivalency in Individual Nanoparticles *via* TIRF Microscopy.* FluMos-v1 nanoparticles were generated as previously described. Nanoparticles were labeled with a Fab cocktail diluted in formulation buffer and consisting of the following fluorescently labeled Fabs: CF405S-F-H1, DyLight550-F-H3, DyLight488-F-HBv, and DyLight650-F-HBy). Following labeling and mixing with FluMos-v1, particles were centrifuged to removed unbound Fab and fluorophores, washed, and further diluted 1:500 in formulation buffer from originally concentration of about 0.07 mg/mL. 200 uL of particle suspension were plated in one well of an ibidi 8-well sticky-Slide (catalog #80828, Gräfelfing, Germany) affixed to an acid-washed #1.5 cover-glass (catalog #152455, Thermo Fisher Scientific, Waltham, MA) and imaged on a Nikon Eclipse Ti2 microscope equipped with an EMCCD iXon Ultra camera (Andor, Oxford, UK) using an SR HP ApoTIRF 1.49 NA, 100× objective. Twenty random fields of view were sequentially imaged with 405, 488, 561, and 640 nm laser lines. Resultant images were analyzed via Nikon Ar Elements software. Images were denoised using the advanced denoising algorithm, then channels were additively combined, and particle locations were determined via spot detection. Local maxima for each separate channel above a threshold determined by examination of singly labeled control nanoparticles were also established via spot detection. Particles were then labeled by valency by comparing particle locations with local channel maxima. Single color localizations with weak signal intensity were excluded from further analysis as dissociated HA trimers, as true monovalent nanoparticles should be highly labeled and exhibit comparatively greater intensities to multivalent particles.

### Supplementary Information

Supplementary Information.

## Data Availability

All data generated or analyzed during this study are included in this published article [and its supplementary information files].
