# Supplementary material for: Mosaic quadrivalent influenza vaccine single nanoparticle characterization
Source: Sci Rep. 2024 Feb 24;14:4534. doi: 10.1038/s41598-024-54876-2 (PMC10894272; doi:10.1038/s41598-024-54876-2)
Supplement: Supplementary file 1 — Supplementary Information. [file 41598_2024_54876_MOESM1_ESM.docx]

Supplementary Materials for

Mosaic Quadrivalent Influenza Vaccine Single Nanoparticle Characterization

Rong Sylvie Yang^1*^, Maria Traver^2*^, Nathan Barefoot^1^, Tyler Stephens^3^, Casper Alabanza^1^, Javier Manzella-Lapeira^2^, Guozhang Zou^1^, Jeremy Wolff^1^, Yile Li^1^, Melissa Resto^1^, William Shadrick^1^, Yanhong Yang^1^, Vera B. Ivleva^1^, Yaroslav Tsybovsky^3^, Kevin Carlton^1^, Joseph Brzostowski^2^, Jason G. Gall^1^, Q. Paula Lei^1^

^1^Vaccine Production Program, Vaccine Research Center, National Institute of Allergy and Infectious Diseases, National Institutes of Health, Gaithersburg, Maryland, USA;

^2^Twinbrook Imaging Facility, LIG, NIAID, NIH

Maryland, USA;

^3^Vaccine Research Center Electron Microscopy Unit, Cancer Research Technology Program, Leidos Biomedical Research, Inc., Frederick National Laboratory for Cancer Research, Frederick, MD, USA

**Corresponding Author:**

Q. Paula Lei

VPP/NIAID/NIH

9 West Watkins Mill Rd.

Gaithersburg, MD, 20878, USA

paula.lei@nih.gov

301-761-7288

^†^These authors contributed equally to the work

**Figure S1:** ELISA assessment of interactions between unlabeled vs. Fluorescently labeled Fab when binding to FluMos-v1 quadrivalent nanoparticle. No impact of fluorophore labeling on the binding interactions with FluMos-v1 for all four Fabs.


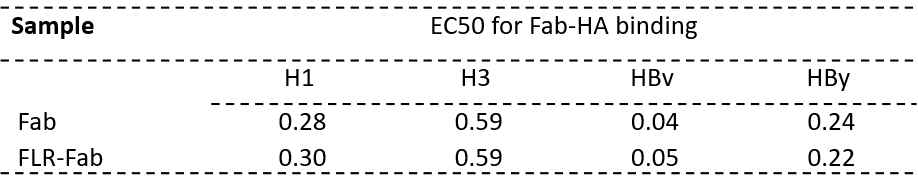


**A**

**B**

**C**

**D**

**Figure S2:** ELISA assessment of MM09 specificity. The four HA trimer proteins were individually tested against the HBy monoclonal antibody MM09.


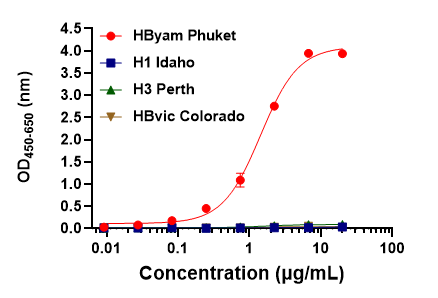


**Figure S3**. Distributions of FLR-labeled Fabs determined by MALDI-MS. The masses of four FLR labeled Fabs (bottom), F-H1, F-H3, F-HBy, and F-HBv were measured by MALDI-MS and compared with the masses of the corresponding non-labeled Fab (top) to estimate the number of the attached FLR labels. The intensities of the peaks shifted due to the labeling were used to calculate the labeling distributions (Table 1). ​

**1 label**

**2 labels**

**F-H1**

**CFS405-F-H1**

**1 label**

**2 labels**

**F-H3**

**D550-F-H3**

**F-HBy**

**1 label**

**D650-F-HBy**

**F-HBv**

**D488-F-HBv**

**1 label**

**2 labels**

**3 labels**

**3 labels**

A

B

C

D

**Figure S4**. Distributions of DyLight-488 labeling of F-H1, F-H3, F-HBv, and F-HBy determined by MALDI-MS. All samples were digested with IgdE except for the samples on the bottom spectrum of panel B, which was digested with papain. (The doubly charged ions are labeled, whereas all other peaks refer to the singly charged ions. The labeled doubly charged Fabs are not annotated.)


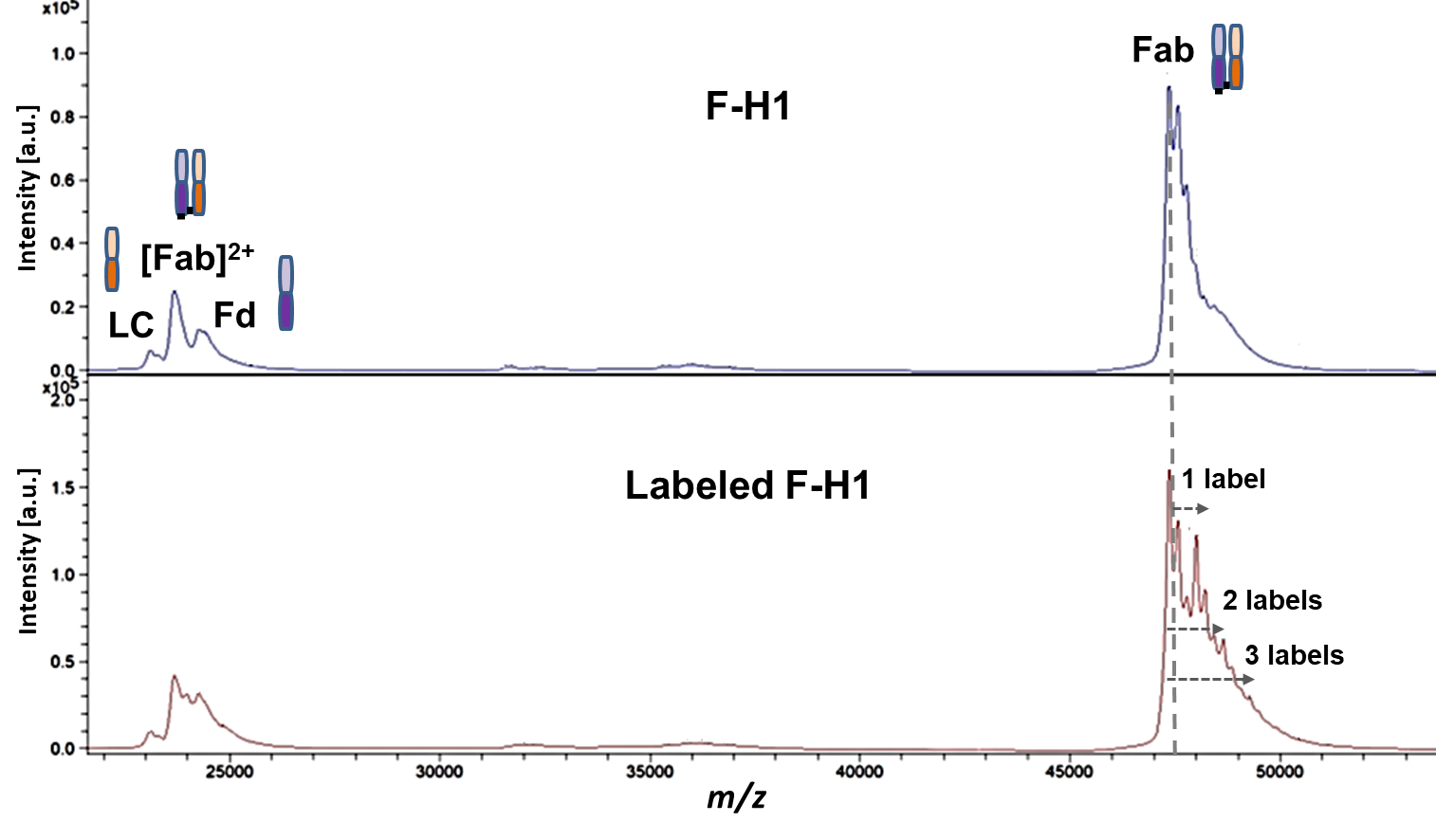

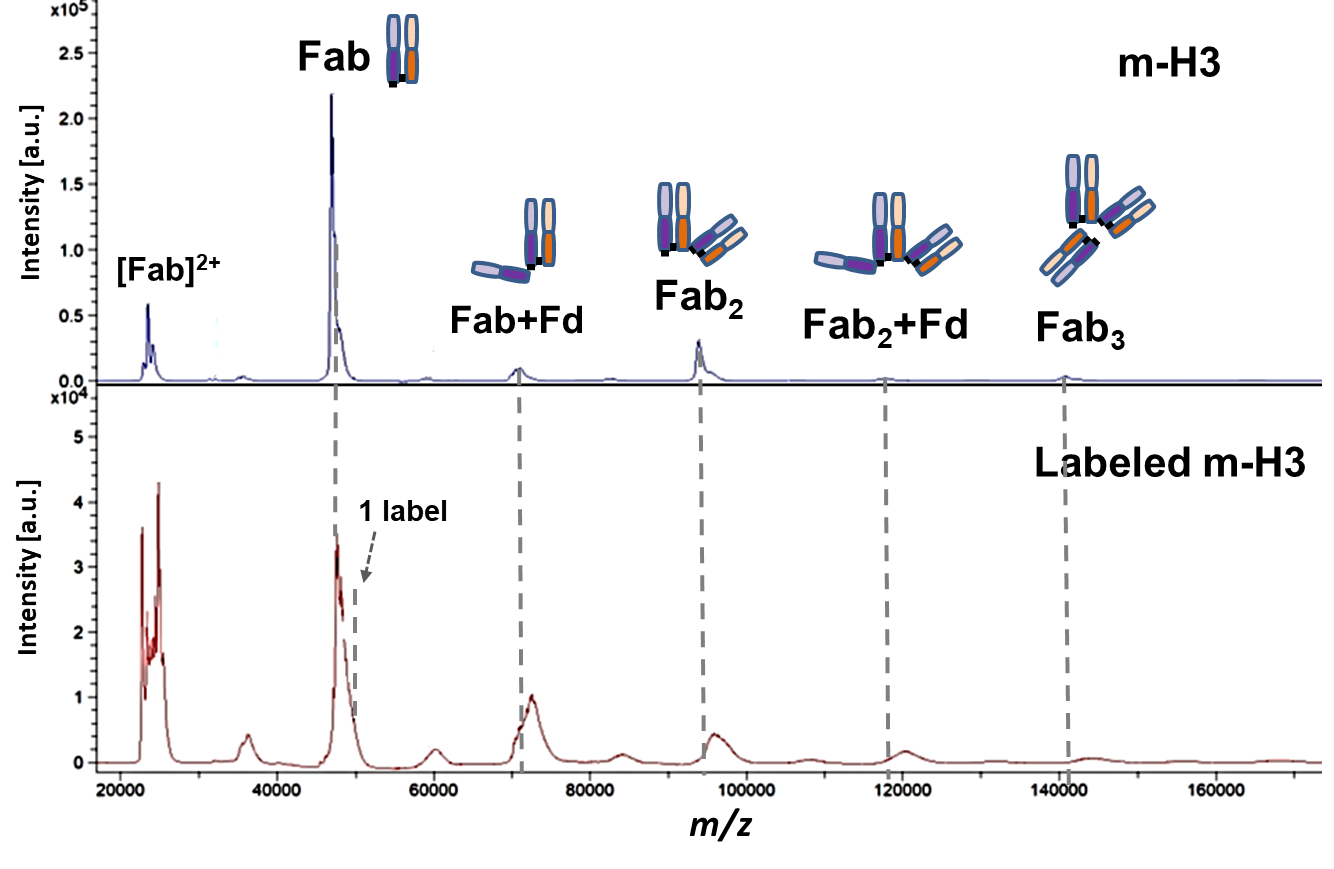

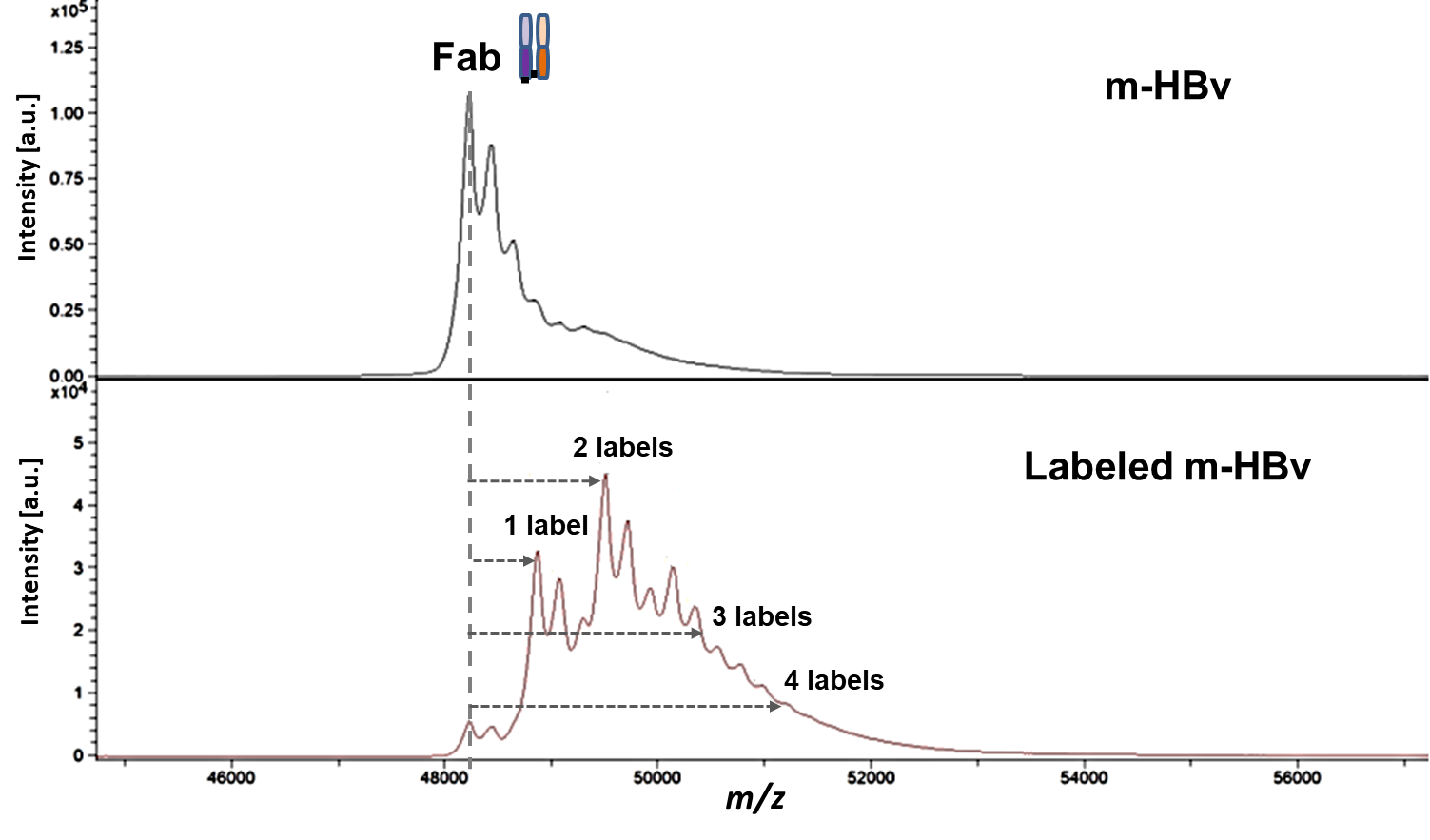

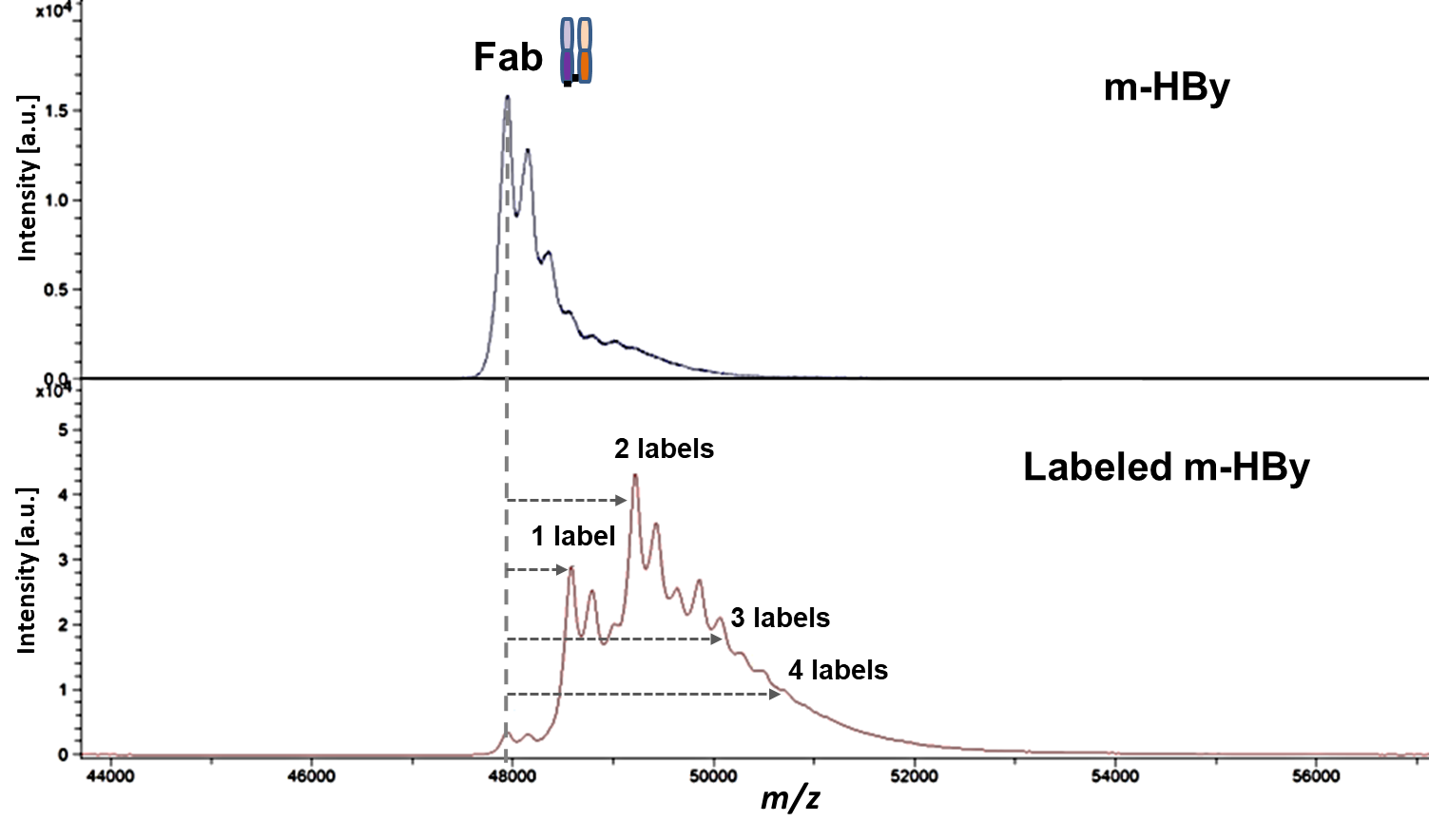


**A**

**B**

**C**

**D**

**Figure S5:** SEC-FLR (intrinsic) chromatogram showing unbound Fab peaks when the amount of Fab exceeds the maximum available binding site of the nanoparticles. This ratio was experimentally confirmed for two HAT strains for two labeled Fabs, F-H1 and F-H3, using unlabeled Fabs and the intrinsic fluorescence of the nanoparticles For monovalent H3- and H1-nanoparticles, extra Fab peak begin to show up at Fab-to-nanoparticle ratio of 60:1. For bivalent H1/H3-nanoparticle, extra Fab peak begin to show at nanoparticle-to-Fab ratio of 40:1. For quadrivalent FluMos-v1, extra Fab peak begin to show at nanoparticle-to-Fab ratio of 15:1 – 20:1.


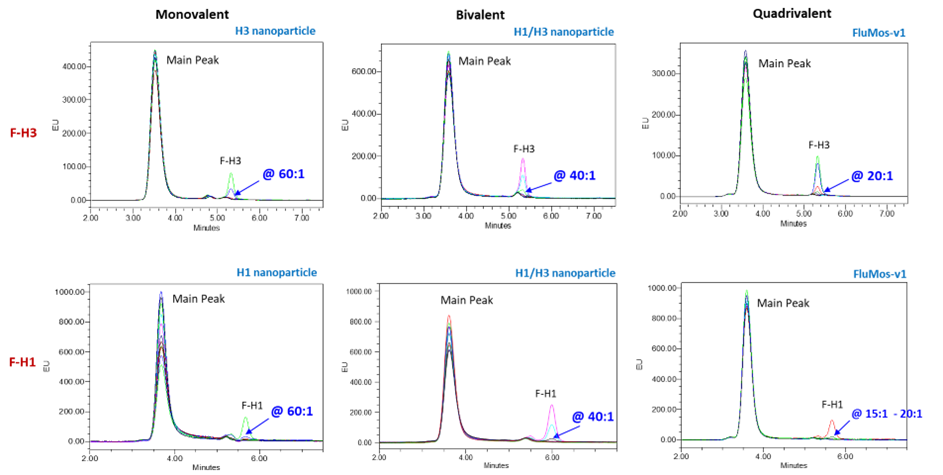


**Figure S6-1:** A TIRF image of labeled monovalent nanoparticles of four types (H1, H3, HBv, and HBy) was obtained by exciting each sample with four wavelengths (405nm, 488nm, 561nm, 640nm). These images serve as a negative control, demonstrating that each label is only excitable by its corresponding wavelength.

**Figure S6-2.** A TIRF image of labeled bivalent nanoparticles composed of H1/H3 was obtained by exciting each sample with two wavelengths (405nm and 561nm).


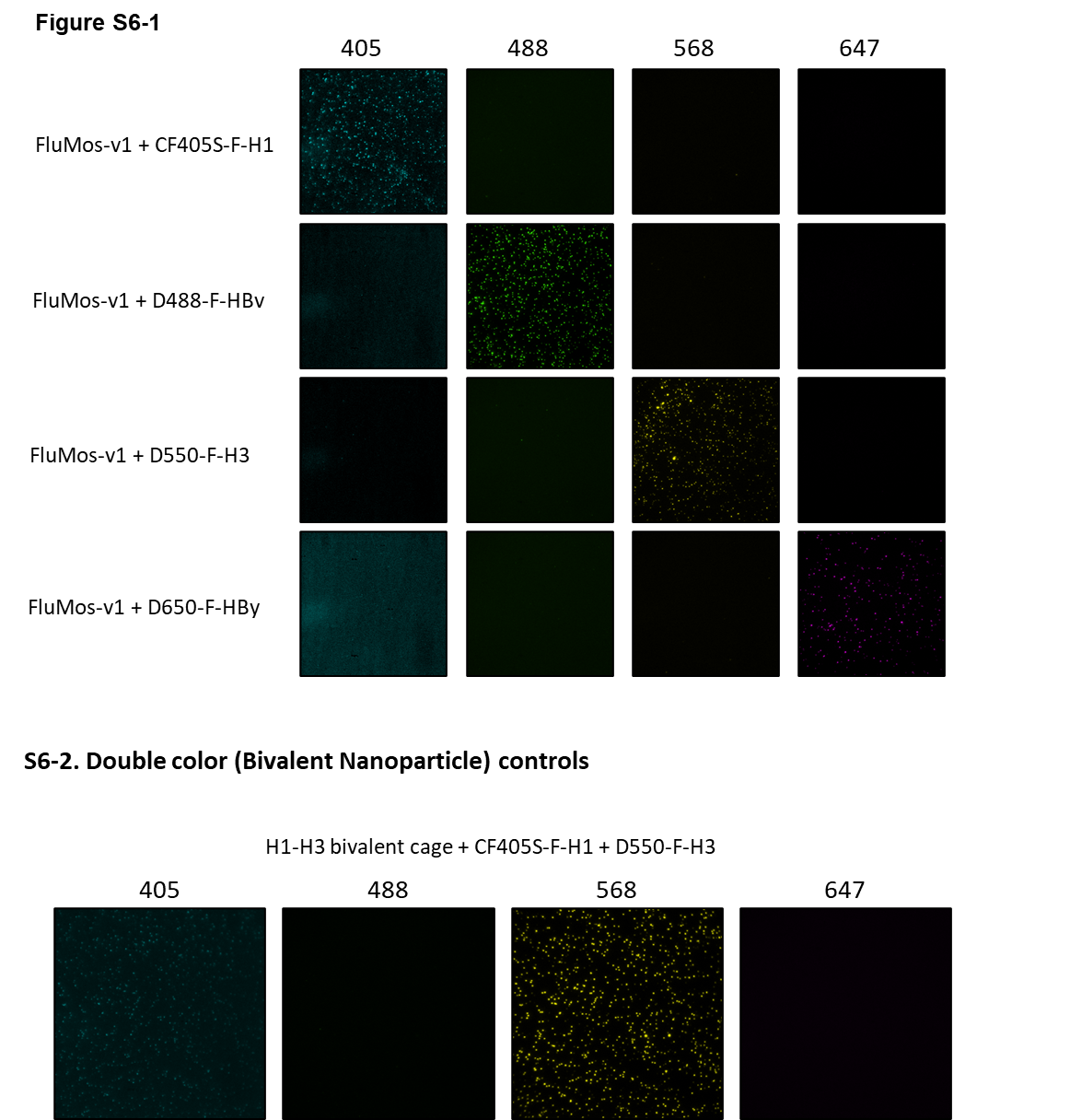


**Table S1**. Theoretical vs. experimental FLR intensity ratio in Monovalent: Bivalent: Tetravalent nanoparticle, demonstrating close to equal molar distribution of 4 HA Trimer (HAT) antigens in single nanoparticles; The ratio was determined by SEC-FLR for all four strains via fluorophore (D488) labeling to individual strain HA(x)’s Fab binding partners: H3, HBv, HBy and H1

| Fabs to be labeled with D488 | Nanoparticles types  (Monovalent): (Bivalent): (Tetravalent) | FLR Intensity Ratio among  monovalent : bivalent : tetravalent nanoparticles |
| --- | --- | --- |
|  |  |  |
| F-H1​ | (H1) : (H1/H3) : (H1/H3/HBv/HBy)​ | 3.7 : 1.7 : 1​ |
|  |  |  |
| F-H3​ | (H3) : (H1/H3) : (H1/H3/HBv/HBy)​ | 3.9 : 2.2 : 1​ |
|  |  |  |
| F-HBv​ | (HBv) : (HBv/HBy) : (H1/H3/HBv/HBy)​ | 4.3 : 2.3 : 1​ |
|  |  |  |
| F-HBy​ | (HBy) : (HBv/HBy) : (H1/H3/HBv/HBy)​ | 5.1 : 2.1 : 1​ |

**Table S2**. Percentage of each population of nanoparticles

|  | Individual Image | | Total Population | |
| --- | --- | --- | --- | --- |
|  | Figure 4A | | Figure 4C | |
| ID | Number of Particles | % | Number of Particles | % |
| H1 + HBv + H3 + HBy | 281 | 43.7 | 7198 | 44.2 |
| H1 + HBv + H3 | 221 | 34.4 | 5185 | 31.9 |
| H1 + HBv +HBy | 6 | 0.9 | 292 | 1.8 |
| H1 + H3 + HBy | 12 | 1.9 | 277 | 1.7 |
| HBv + H3 + HBy | 53 | 8.2 | 1349 | 8.3 |
| H3 + HBy | 2 | 0.3 | 124 | 0.8 |
| HBv + HBy | 1 | 0.2 | 89 | 0.6 |
| HBv + H3 | 42 | 6.5 | 1129 | 6.9 |
| H1 + HBy | 1 | 0.2 | 56 | 0.3 |
| H1 + H3 | 5 | 0.8 | 201 | 1.2 |
| H1 + HBv | 16 | 2.4 | 274 | 1.7 |
| HBy | 0 | 0 | 4 | 0 |
| H3 | 2 | 0.3 | 54 | 0.3 |
| HBv | 1 | 0.2 | 41 | 0.3 |
| H1 | 0 | 0 | 0 | 0 |
|  |  |  |  |  |
| Total | 643 | 100 | 16273 | 100 |
